# The future of digital donation crowdfunding

**DOI:** 10.1371/journal.pone.0275898

**Published:** 2022-11-11

**Authors:** Siriphong Sirisawat, Pattanaporn Chatjuthamard, Supaporn Kiattisin, Sirimon Treepongkaruna

**Affiliations:** 1 Faculty of Engineering, Mahidol University, Nakorn Pathom, Thailand; 2 Center of Excellence in Management Research for Corporate Governance and Behavioral, Sasin School of Management, Chulalongkorn University, Bangkok, Thailand; 3 Sustainability in Financial and Capital Market Research Unit, Sasin School of Management, Chulalongkorn University, Bangkok, Thailand; 4 UWA Business School, University of Western Australia, Perth, Australia; University of Pisa, ITALY

## Abstract

Amidst the Coronavirus crisis, many fundraising projects have emerged to relieve financial burdens resulting from social distancing policies. Crowdfunding is a way to raise money to fund a business, project or charity, through either social media or other online platforms to reach hundreds of potential sponsors. We developed guidelines for effective donation-based crowdfunding through online platforms. Using Futures Research (FR) technique, we conducted our analyses in 3 phases. In Phase 1, we reviewed relevant literature and conducted in-depth interviews of related parties. In Phase 2, we interviewed experts using Ethnographic Futures Research (EFR) technique. In Phase 3, we visualized the future using the principles of Futures Wheel, Cross-impact Matrix and Scenarios. Based on our findings, effective donation-based crowdfunding platforms should adopt Blockchain technology for transparency and accountability, and incentivize donations to keep backers loyal. Founders should be required to obtain fundraising licenses from relevant regulators. Finally, laws and regulations that protect platform users should be standardized internationally. Our proposed guidelines hope to improve the quality and transparency of future fundraising activities.

## Introduction

The current pandemic resulted from the spread of a strain of Coronavirus, officially known as Coronavirus 2019 (COVID-19), that began in China and has spread around the world. Since the year 2000, we have seen the emergence of a novel disease that has resulted in large-scale contagion at least once every decade, namely the SARS epidemic in 2002–2003, the swine flu pandemic in 2009–2010, the Ebola epidemic in 2014–2016, and the MERS epidemic from 2015 –present reported by Nicholas [1]. Since 2019 COVID-19 has created mass global panic and led to unprecedented public concern over cash spending [2]. Research has shown that infection can be transmitted through contact with banknotes and coins [3,4] as well as credit cards, debit cards and cell phones [2,5]. Experts from the World Health Organization (WHO) advise people to wash their hands after touching cash or other objects to help reduce their risk of contracting the virus [6]. People’s behavior towards spending money has changed and has increasingly turned to spending via digital channels [5].

Despite the virulence of each plague, one thing that represents the psychological beauty of humanity is that people raise money to help others who have been affected by the incident, with donations being raised through online social platforms such as JustGiving, GoFundMe and Taejai. Most donations are made via e-payment channels according to the Global NGO technology report (NGOs) in 2019, which found that 64% of NGOs worldwide accept online donations on their website through payment methods such as credit cards, direct debit, PayPal, digital wallet and bitcoin [7]. However, fundraising through online crowdfunding platforms are subject to many requirements and restrictions. Founders must propose their project to the platform’s board, which may take 3–7 days to approve. Upon approval, most platforms charge a backers’ fees totaling 3–12% of donations received by the platform. The founders’ geographical location may also impact the currency in which their fundraising can take place, and international money transfer fees. For further information, donors can find out about fundraising programs through the platform and make donations via channels such as credit, debit, PayPal from website information [8–11]. The 2019 Global NGO technology report suggests that 90% of sample use social media to engage their supporters and donors, with the most popular social media platforms being Facebook, Twitter, Linkedln, WhatsApp, and YouTube, respectively [7]. Even during the COVID-19 crisis, Facebook created a tool to raise donations in some countries without fees[12] Fundraising through social channels can be done quickly because it does not have to be approved by the platform’s board. However, by transferring money directly via social media, donors are subject to risk of misrepresentation due to lack of information about fundraisers [13]. This leads us to explore stakeholder characteristics required to make a successful donation-based crowdfunding platform using the principles of Futures Wheel.

Thus, this research studies the potential direction for the development and improvement of donation crowdfunding platforms that will meet needs of stakeholders now and in the future. We identify key success factors, which can act as practical guidelines for each stakeholder group.

## Review of related literature

### Futures research

According to Jerome [14] & Roy [15], Futures Research (FR) is a systematic approach to find answers to future choices by considering the reasonable possibilities that are the outcome of recent decisions and policies. Futures research is long-term planning consisting of three activities: planning, forecasting, and decision-making. The aim of futures research is to negate the preconceived notion that the future is a fantasy, but rather that it is predictable and possible. The heart of futures research is to expand thinking from simple predictions, to exploring, conceptualizing, and testing desirable future outcomes. This vision of the future will help shaping long-term policies, tactics, and planning that leads to achieving what we need in the future. It uses a group of relevant experts as the stakeholders while determining the future factor composition guidelines.

### Donation-based crowdfunding & crowdfunding ecosystems

There exists four key types of crowdfunding: 1) donation-based crowdfunding, 2) reward-based crowdfunding, 3) lending-based crowdfunding, and 4) equity-based crowdfunding [16,17]. This paper focuses only on donation-based crowdfunding. We define a donation-based crowdfunding platform as a technological intermediary used by fundraisers to match donations with donors’ objectives, which are not monetary, but psychological ones. Following Beaulieu et al. [17], we identify stakeholders in crowdfunding ecosystems as those shown in Table 1. We summarize existing literature on specific success factors and components of donation-based crowdfunding in Table 2. Overall, stakeholders of donation-based crowdfunding are not those in the traditional capital markets involved in other types of crowdfunding such as reward-based crowdfunding, lending-based crowdfunding, and equity-based crowdfunding.

**Table 1:** Stakeholders of crowdfunding ecosystems.

Stakeholder	Description
Website providers	Provide web services and build a system to support the work of founders in presenting their projects to potential backers for raising donations. In addition, they must create a system for managing users’ information that is user-friendly, allows feedback from stakeholders, allows sharing through social media, secure, transparent, and adaptable to future technology.
Founders	Individuals or groups who establish and present projects on crowdfunding sites to raise donations for stipulated project goals.
Backers	In addition to donating funds, they also assess fundraising projects to identify those that they deem worthwhile and share information to promote those projects through social media or their personal networks.
Traditional Capital Markets	Source of funds for backers and founders, such as angels, VC funds, and banks, etc.
Legal/Ethical	Laws and regulations governing the crowdfunding activities to ensure safety and fairness to all stakeholders. However, it is important to consider the laws governing crowdfunding are not uniform across the globe due to differences in racial, social, and cultural contexts. Therefore, we must also apply ethical principles, analysis, and due diligence in reviewing projects before implementation and funding.

**Table 2:** The factors of the stakeholders of the donation crowdfunding system.

Stakeholders	Factor	Reference
Website providers	System development	Tanya Beaulieu, et al. [18] Kevin J. Boudreau, et al. [19] Ivo Jenik, et al. [20] Jaya Gera, et al. [21] Emel Filiz-Ozbay, et al. [22] Arief Rijanto [17] Shusaku Sasaki [23] Karina Sokolova and Charles Perez [24] Yuangao Chen, et al. [25] Michele Scataglini and Marc J Ventresca [26] Abhishek Behl, et al. [27] Yali Zhang, et al. [28] Hongke Zhao, et al. [29]
	Technology	Tanya Beaulieu, et al. [18] Ivo Jenik, et al. [20] Jaya Gera, et al. [21] Emel Filiz-Ozbay, et al. [22] Arief Rijanto [17] Karina Sokolova and Charles Perez [24] Michele Scataglini and Marc J Ventresca [26]
	Social partner	Ivo Jenik, et al. [20] Jaya Gera, et al. [21] Emel Filiz-Ozbay, et al. [22] Larry Zhiming Xu [30] Karina Sokolova and Charles Perez [24] Michele Scataglini and Marc J Ventresca [26]
Founders	Founder experience	Tanya Beaulieu, et al. [18] Kevin J. Boudreau, et al. [19] Nianxin Wang, et al. [31] Jaya Gera, et al. [21] Hui-Ching Hsieh, et al. [32] Michele Scataglini and Marc J Ventresca [26] Rotem Shneor, et al. [33]
	Founder characteristic	Tanya Beaulieu, et al. [18] Kevin J. Boudreau, et al. [19] Jaya Gera, et al. [21] Emel Filiz-Ozbay, et al. [22] Rotem Shneor, et al. [33] Shusaku Sasaki [23]
	Project/Campaign	Tanya Beaulieu, et al. [18] Kevin J. Boudreau, et al. [19] Arief Rijanto. [17] Hui-Ching Hsieh, et al. [32] Larry Zhiming Xu [30] Karina Sokolova and Charles Perez [24] Yuangao Chen, et al. [25] Gaia Bassani, et al. [34] Abhishek Behl, et al. [27] Rotem Shneor, et al. [33] Michele Scataglini and Marc J Ventresca [26]
	Motivation	Tanya Beaulieu, et al. [18] Kevin J. Boudreau, et al. [19] Ivo Jenik, et al. [20] EmelFiliz-Ozbay, et al. [22] Timothy N.Cason and RobertasZubrickasb [35] AriefRijanto [17] Shusaku Sasaki [23] Larry Zhiming Xu [30] Yuangao Chen, et al. [25] Gaia Bas-sani, et al. [34] Yali Zhang, et al. [28] RotemShneor, et al. [33] Hongke Zhao, et al. [29]
	Satisfaction	Ivo Jenik, et al. [20] Arief Rijanto [17] Shusaku Sasaki [23] Yuangao Chen, et al. [25]
	Tracking	Ivo Jenik, et al. [20] Shusaku Sasaki [23]
Legal	Law	Tanya Beaulieu, et al. [18] Ivo Jenik, et al. [20] Michele Scataglini and Marc J Ventresca [26] Yali Zhang, et al. [28]
	Rules	Ivo Jenik, et al. [20] Alice Rossi and Silvio Vismara [36] Michele Scataglini and Marc J Ventresca [26]
	Ethics	Tanya Beaulieu, et al. [18]

## Methods

### Futures research

**Fig 1:**
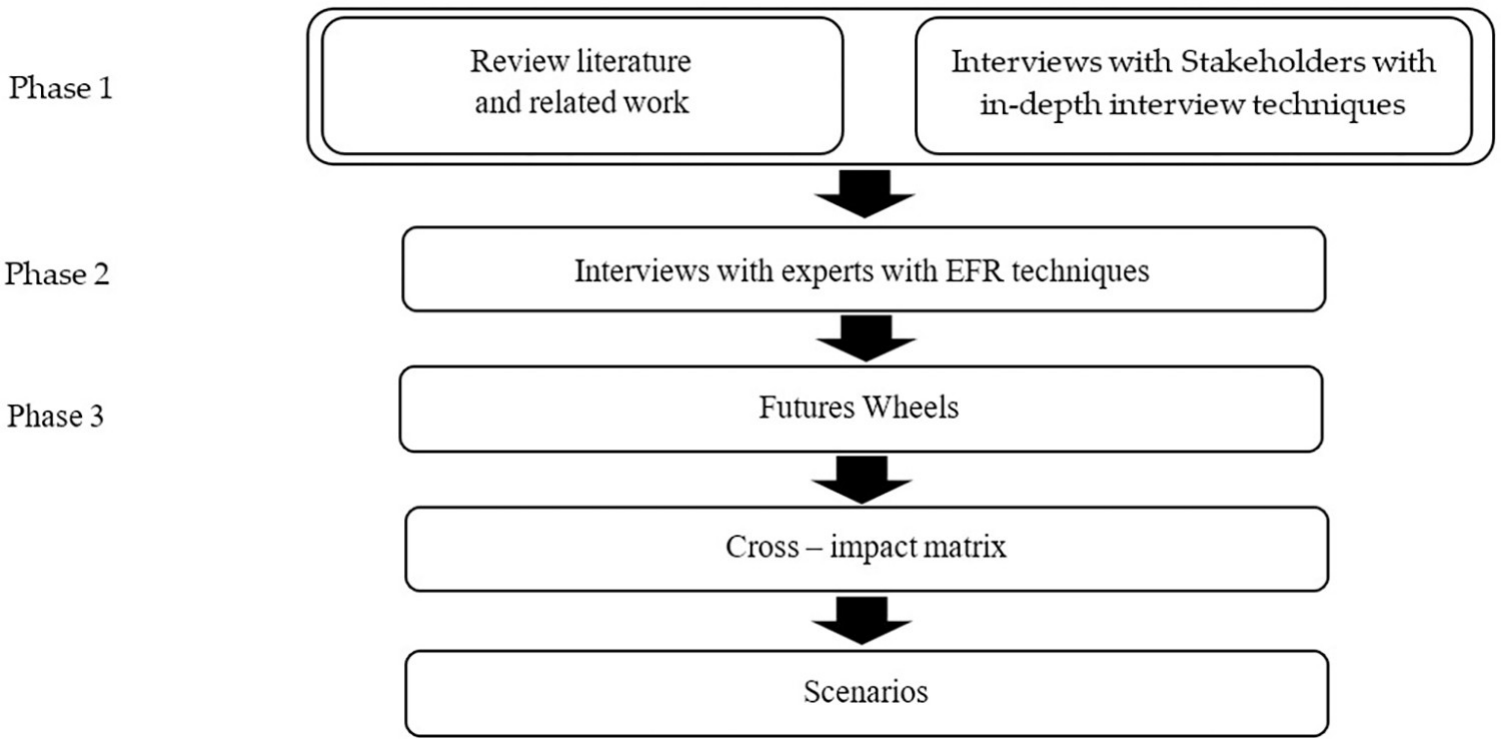
Three phases of our analysis.

To study the future of crowdfunding donations, we use Futures Research techniques which can be divided into normative and exploratory forecasting [14]. This allows us to develop a donation-based crowdfunding platform by letting stakeholders determine the direction, composition, and success factors of the platform. Fig 1 depicts 3 phases of our analysis as follows.

Phase 1: We collected fundamental data by reviewing existing literature. We then interviewed relevant stakeholders using in-depth interview techniques to obtain characteristics and components of donation-based crowdfunding in addition to those obtained through literature review.

Phase 2: We interviewed experts and qualified persons selected using Ethnographic Futures Research techniques aimed at classification, narrative, analysis, interpretation and image diagnosis. Experts and other interviewees were checked to ensure they possess required qualifications to be in our study prior to the interview appointment. Interviewees were allowed to change the interview structure by adding or excluding certain topics as they saw fit during the interview. The interviewer thoroughly listened to the content and took notes for further analysis [14,15]. The interviewees were offered three possible future scenarios: 1) optimistic-realistic scenario, 2) pessimistic-realistic scenario, and 3) the most-probable scenario. Based on interviewee’s choice, we obtained the attributes and elements of successful donation-based crowdfunding.

Phase 3: The Futures Wheel, a participatory ‘‘smart group” method, utilizes structured brainstorming process to reveal multiple levels of consequences arising from changes. After interviewing experts, we drafted the possible futures of donation-based crowdfunding by creating a futures wheel, analyzing cross-impact matrix sourced from experts in phase 2, and describing envisioned future donation crowdfunding scenarios resulting from research.

### Subject selection and allocation

To select and allocate subject, we used purposive sampling method as follows.

Group 1 We conducted in-depth interviews with 21 stakeholders to understand the current fundraising system as shown in Table 3.

**Table 3:** Stakeholders’ data in group 1 interviews.

Group	Stakeholders	Total
Website provider	1) Entrepreneurs or developers of fundraising platforms in Thailand. 2) Technological experts.	6 individuals
Founders	1) Donor fundraisers who previously raised funds from fundraising platforms. 2) Donors who have previously raised funds through social media.	6 individuals
Backers	1) Donors who have donated through fundraising platforms. 2) Donors who have donated through the social media.	6 individuals
Lawyers	Persons who have a degree in the law and provide sound guidance.	3 individuals
		21 people

Group 2 We interviewed 20 experts with knowledge and experience in fundraising for donation-based crowdfunding as shown in Table 4.

**Table 4:** Information of experts in group 2 interviews.

No.	Group of Experts	Total
1	Individuals from government agencies in charge of raising donations from the Securities and Exchange Commission (SEC) and the Ministry of Interior in Thailand.	2 individuals
2	1) Developer of fundraising platform in Thailand. 2) Entrepreneurs raising funds for donations in Thailand. 3) Developer of a cryptocurrency-based fundraising platform.	6 individuals
3	Successful endowment fundraisers who have raised funds from donation-based fundraising platforms and social media in Thailand.	3 individuals
4	Experienced donors donating more than 10 times through donation fundraising and social media platforms in Thailand.	3 individuals
5	Technologists from the Ministry of Digital Economy and Society and technological experts from Thai universities.	3 individuals
6	Individuals who oversee the legal framework for fundraising and related laws from the SEC, Ministry of Justice, and Ministry of Digital Economy and Society of Thailand.	3 individuals
		20 people

Applying Macmillan [36], we select number of experts within the range of 17–21 with a 0.5 margin of error.

### Data collection process

As our study is human subject research, we sought required approval from the Institutional Review Board (IRB) to conduct this research. After receiving this approval, we began collecting data through interviews with stakeholders and experts, taking notes and voice recordings. Interview lengths range from 45 to 60 minutes. We then utilised data collected to create a Futures Wheel and conducted a cross matrix scenario analysis.

## Results

### Sketching the future of donation-based crowdfunding

**Fig 2:**
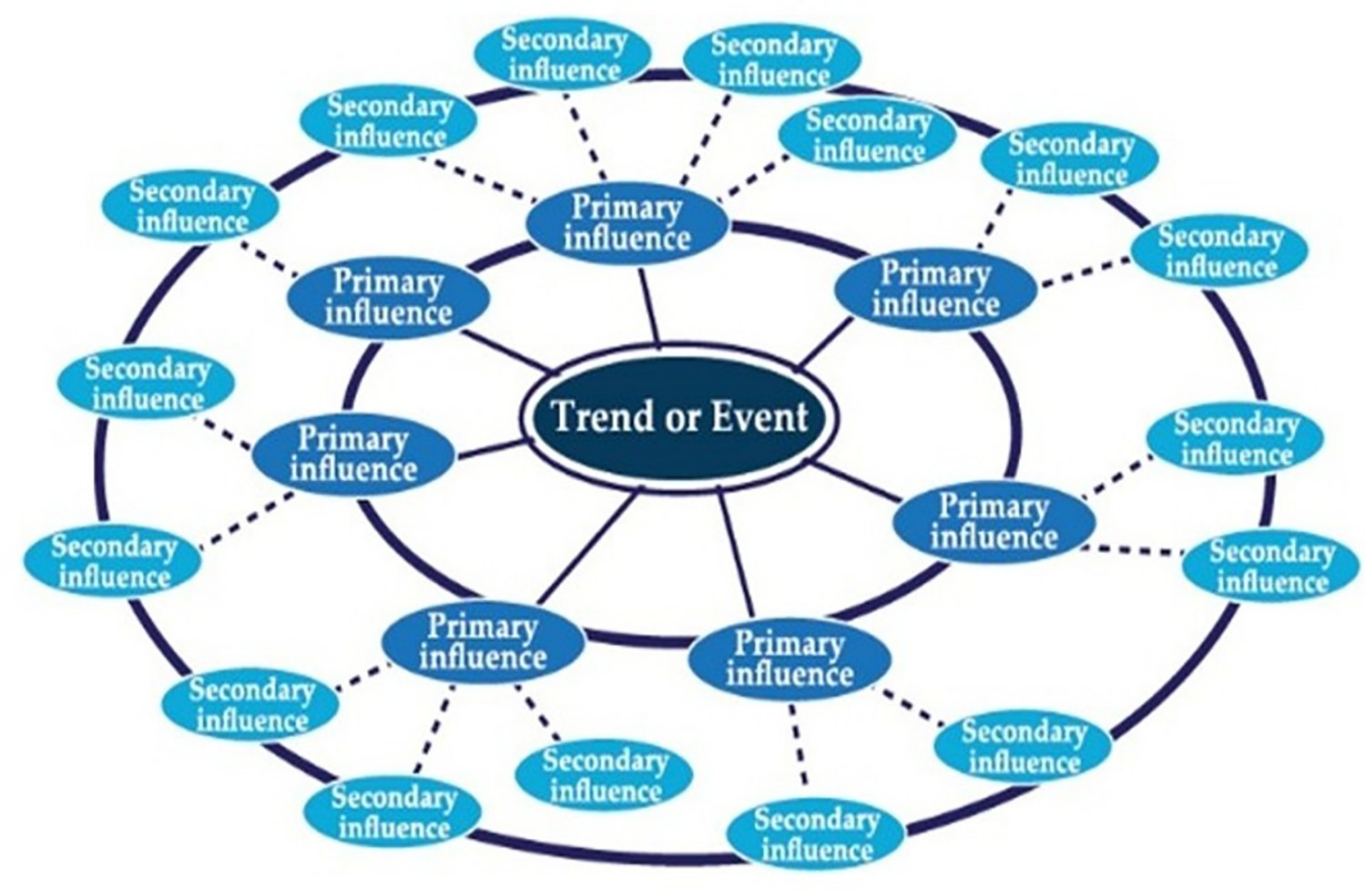
Future donation crowdfunding model synthesis by stakeholder group.

**Fig 3:**
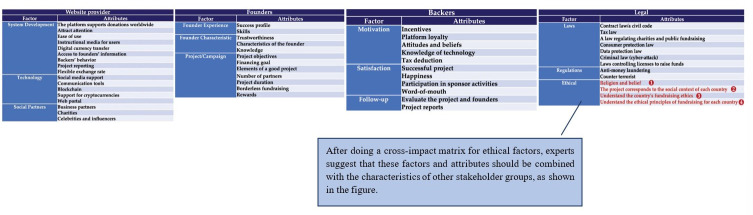
Future donation crowdfunding model synthesis by stakeholder group.

**Fig 4:**
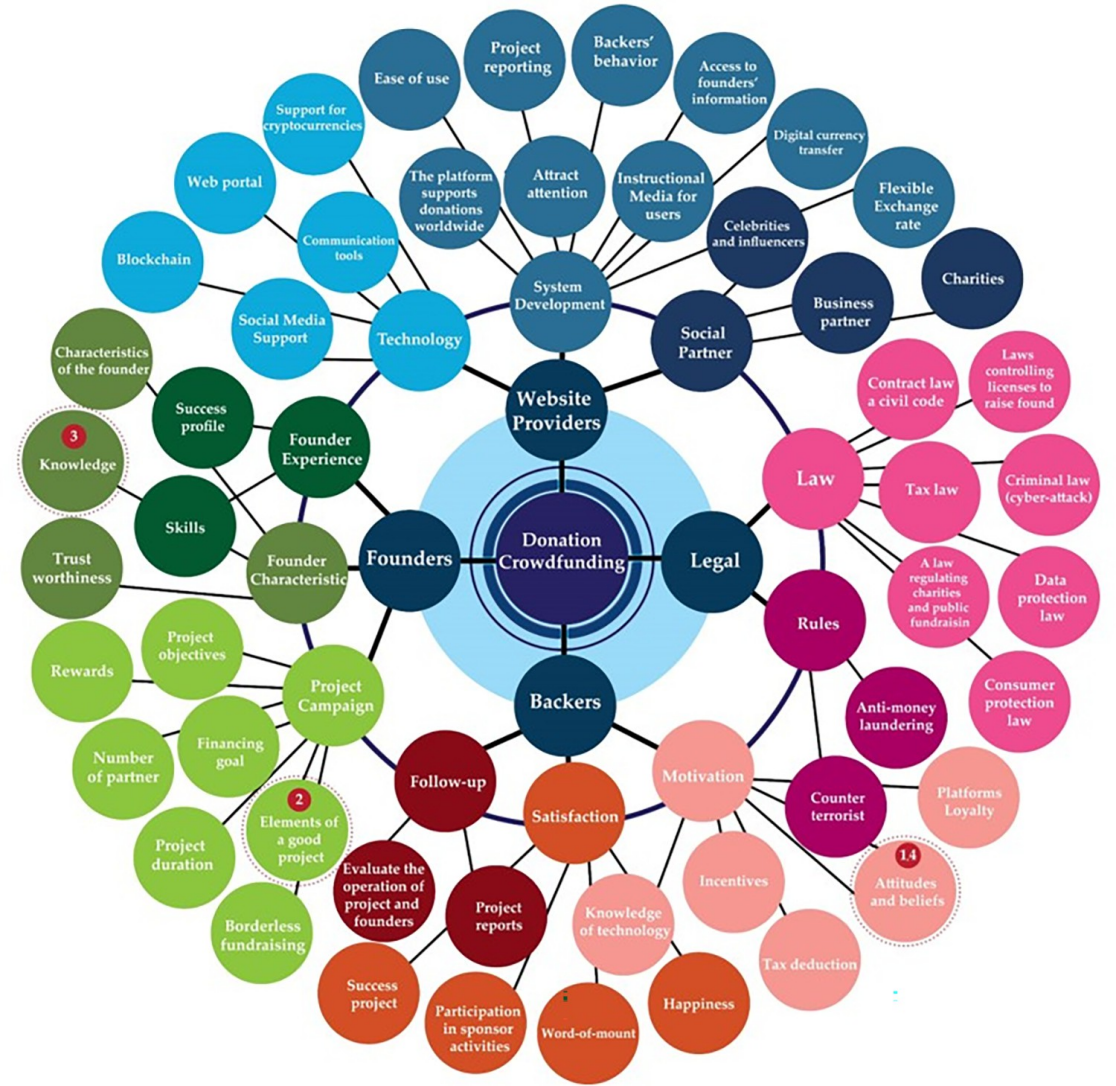
Future donation crowdfunding model synthesis by stakeholder group.

Using the principles of the Futures Wheel, we asked a group of experts to evaluate consequences for each scenario in the cross-impact matrix to determine the relationships and interdependencies among compositions and factors of donation-based crowdfunding shown in Figs 2–4.

We rate the significance of each relationship using a three-level scale, where +1 is highly relevant, 1 is somewhat relevant, and 0 is not relevant. These scores are generated from the assessment of 20 experts involved in our research. Factors that are deemed by more than 70% of experts as relevant are given a score of +1 and included in Tables 5–8 below.

**Table 5:** Expert assessment (N = 20) of attributes relevant to the website provider group in future development of donation crowdfunding platforms.

Factor	Value (+1)	Percentage
System development		
The platform supports donations worldwide	20	100
Attract attention	20	100
Ease of use	20	100
Instructional media for users	20	100
Digital currency transfer	19	95
Access to founders’information	20	100
Backers’ behavior	18	90
Project reporting	20	100
Flexible exchange rate	19	95
Technology		
Social media support	20	100
Communication tools	20	100
Blockchain	19	95
Support for cryptocurrencies	19	95
Web portal	18	90
Social Partners		
Business partners	20	100
Charities	20	100
Celebrities and influencers	20	100

**Table 6:** Expert assessment (N = 20) of attributes relevant to the founders’ group in future development of donation crowdfunding platforms.

Factor	Value (+1)	Percentage
Founder experience		
Success profile	20	100
Skills	20	100
Founder characteristic		
Trustworthiness	18	90
Characteristics of the founder	18	90
Knowledge	18	90
Project/Campaign		
Project objectives	20	100
Financing goal	16	80
Elements of a good project	20	100
Number of partners	18	90
Project duration	18	90
Borderless fundraising	16	80
Rewards	14	70

**Table 7:** Expert assessment (N = 20) of attributes relevant to the backers’ group in future development of donation crowdfunding platforms.

Factor	Value (+1)	Percentage
Motivation		
Incentives	20	100
Platform loyalty	20	100
Attitudes and beliefs	18	90
Knowledge of technology	17	85
Tax deduction	14	70
Satisfaction		
Successful project	18	90
Happiness	17	85
Participation in sponsor activities	18	90
Word-of-mouth	14	70
Follow-up		
Evaluate the project and founders	20	100
Project reports	18	90

**Table 8:** Expert assessment (N = 20) of attributes relevant to the legal group in future development of donation crowdfunding platforms.

Factor	Value (+1)	Percentage
Laws		
Contract law/a civil code	18	90
Tax law	20	100
A law regulating charities and public fundraising	20	100
Consumer protection law	20	100
Data protection law	18	90
Criminal law (Cyber-attack)	20	100
Laws controlling licenses to raise funds	20	100
Regulations		
Anti-money laundering	20	100
Counter terrorist	20	100

### Findings related to the composition and factors of stakeholders on the future of donation-based crowdfunding and scenario

Based on the analysis of the composition and factors of stakeholders, successful donation-based crowdfunding of the future should comprise of the following groups of stakeholders. Each group of stakeholders has its own scenario.

### Website providers

Scenario “Website providers should develop a platform that supports global donations, incorporates technology such as Blockchain, and considers UX/UI to ensure it is secure, easy to use and satisfying for the next generation of users.”

Website providers should develop a donation-based crowdfunding platform by taking into account 3 main factors: 1) System Development 2) Technology 3) Social Partner. Details are shown in Table 9.

**Table 9:** The factors and details of the website provider group composition.

Factor	Attributes	Description
System development	The platform supports donations worldwide	Donation crowdfunding platforms should be able to support donations around the world without geographical and currency restrictions, possibly through the use of Blockchain-based business models. Blockchain systems have the potential to decentralize business operations, transform how transactions occur, facilitate direct access to large amounts of stakeholders’ data, and most importantly, connect and track users to increase transparency in platform operations.
	Attract attention	To attract attention, the platform business model should incorporate the Hook model of behavioral design. This is a user engagement framework that works to encourage users to actively and repetitively engage in a desired activity or interact with the platform. The model includes the following 4 stages: trigger, action, variable reward and investment, which can be used to customize the platform for the aforementioned stakeholder groups. 1) Triggers are potential motivators that can spark interest in the platform. They are divided into two types: 1.1 Internal triggers are the emotions and desires of users. For founders, this could be the feeling that the platform is easy to use and convenient. For backers, this could be the feelings of sympathy, compassion, fulfillment, or the desire to help victims of disasters. 1.2 External triggers are prompts created by the platform, such as images, videos, call to action buttons, notifications, and reminders. Platforms should push like, comments, tags and other notifications. These can remind stakeholders to stay interested and use the platform. 2) The target action should be as simple and quick as possible. For example, to encourage Founders to register, they should be able to seamlessly fill out information online, create funding arrangements through Blockchain smart contracts, and monitor real-time fundraising results on the platform. Backers should receive project suggestions as soon as they sign up and provide information on their interests. They should also be able to opt into receiving notifications on projects they wish to follow. 3) Rewards can be divided into 3 types: 3.1 Social reward is satisfaction gained from being recognized by others. For example, founders may derive this satisfaction from being recognized as a top fundraiser by the platform or having a number of people follow their projects. Conversely, backers can also be recognized by the platform as a top tier donor and share their contributions through social media. 3.2 Resources satisfaction is the satisfaction the user gains from accessing the platform’s resources. For example, founders can raise funds and learn about other projects. Backers can track their favorite founders and their future projects. 3.3 Self-achievement is the satisfaction gained from own success and improvement. Founders may gain this sense of achievement by receiving high performance ratings from backers or becoming a top fundraiser on the platform. Backers, on the other hand, may feel self-achievement from being recognized as a premium privilege member based on the number and amount of donations made to projects on the platform. 4) Users’ investment is what users give back to the platform, namely, continuous usage. A good platform should be designed to allow users to also contribute to the platform, as this will increase their bond with it. For example, after successfully raising funds for their projects, founders may also donate to other projects and attract new backers to the platform. Backers may feel satisfied with the outcomes of projects they’ve supported and share their positive experience through social or word of mouth. This would increase support for projects and consequentially the number of users on the platform. Website providers should apply the principles of User Experience Design (UXD) in designing a platform that satisfies its users. Users must be able to complete all necessary funding tasks on the platform. This makes is more likely that they will continue to engage with the platform in the future.
	Ease of use	1. Website Providers should apply the principles of UI design in designing the platform webpage, incorporating visual aids, such as buttons and icons, and user guides that are easily understood. 2. The platform should contain an interactive chat tool that enables communication between all system stakeholders, with a chatbot to answer frequently asked questions about the system. This makes for a more convenient and faster user experience. Most importantly, the platform should support automated performance notifications and project evaluations through social media and mobile application.
	Instructional media for users	The platform should have a digital and interactive user manual to enable new founders and backers to learn the usage procedures and rules of use of the platform. The use of Game-Based Learning techniques with rewards can motivate users to complete learning materials and engage with the platform. Rewards may include digital currency that can be donated to a project of their choice.
	Digital currency transfer	The platform should accept donations of all cryptocurrencies. They should also automatically convert donations received into the currencies required by each project.
	Access to Founders’ information	The platform should allow users to manage their own information, including basic data, project data, donation data, and evaluation data.
	Backers’ behavior	The platform should use artificial intelligence to learn about the behaviors of individual donors to allow them to suggest projects that are likely to be suitable for each donor based on known interests and current trends.
	Project reporting	The platform should provide information on the founders and their projects. The platform should have a system for reporting to backers about the progress of projects they’ve supported from beginning to end. Project transparency can build backers’ trust in the projects and enhance platform credibility. Most importantly, it can help backers and the platform verify project information to prevent fraud and exploitation.
	Flexible exchange rate	The platform should have a system to automatically manage donation and withholding tax fees.
Technology	Social media support	Platform development should be compatible with social media and mobile applications, especially when entering the 6G-enabled mobile edge computation era in the near future. Social media may even be used as a fundraising platform.
	Communication tools	The platform should support easy connection to other applications through one click, such as being able to promote the fundraising project information via social media Live tools, and sharing project information to applications such as Facebook, YouTube and TikTok, etc. During a Live, viewers should be able to instantaneously make a donation in their desired currency, including digital currency, through automatic conversion by the platform. Another potential communication channel for raising funds may be e-commerce platforms, including Lazada, Shopee, and Alibaba.
	Blockchain	Blockchain and Smart Contract should be used in platform development to facilitate fundraising contracts for founders and backers to eliminate transfer fees and reduce the geographic limitations of fundraising. In addition, with increasing prevalence of 6G, we expect that Blockchain will have more practical and efficient functions.
	Support for cryptocurrencies	The platform should develop a system capable of supporting multiple cryptocurrencies.
	Web portal	The platform should be developed in the form of a web portal.
Social partners	Business partners	The platform should collaborate with parties that can assist with promoting and supporting fundraising efforts, such as social media and e-commerce platforms. These partners may help produce digital coins that can be used for donations instead of cash. Having reputable business partners gives potential donors increased confidence in the donation projects and the platform.
	Charities	The platform should work with charities, as they have existing infrastructure that can be used for fundraising activities and even resources to donate to projects. Founders may be able to choose charities with consistent objectives to support or promote their project. The founders may also directly send requests to charities so that they can approve projects through the platform.
	Celebrities and influencers	The platform should have a network with celebrities and influencers to help with promoting the fundraising platform and connecting them with founders on specific projects that are aligned with their objectives. They can support the project or assist with providing publicity.

### Founders

Scenario “Founders in the future will be required to have a legal fundraising license, skills and experience in fundraising, good attributes, and offer creative projects that meet societal needs.”

Good founders in the future is defined by three main factors: 1) Founder experience 2) Founder characteristics, and 3) Project/Campaign. Details of each factor including sub-components are shown in Table 10.

**Table 10:** The factors and details of the founders group composition. An attribute with less than 70% relevance score is Market Share under the Project/Campaign category, which considers the uniqueness of a project’s objectives compared to others. If a project seems similar to existing projects, founders should try to identify ways to differentiate it in order to increase social value and fundraising success.

Factor	Attributes	Description
Founder experience	Success profile	Past performance of fundraising projects and fundraising achievements.
	Skills	Good fundraising management skills.
Founder characteristic	Trustworthiness	1. Founders must have a donation fundraising license from the government or an organization authorized by the government to issue fundraising licenses. 2. Founders must have a good social reputation, a team to operate the fundraising project, and partner with social networks to raise funds.
	Characteristics of the founder	Honesty and enthusiasm for funding projects, such as always reporting on project progress and movements, and importantly, always answering problems and questions in fundraising from donors, or having a team to answer questions including chatrooms to build trust with donors.
	Knowledge	Founders should have technological knowledge required to use the platform and associated applications. Founders should have knowledge of 2 languages or more, especially commonly spoken languages such as English. Significantly, they should also have a good understanding of the attitudes, beliefs, religions, and ethics of regions where they’ll be conducting fundraising activities to ensure they can communicate in a way that is respectful.
Project/Campaign	Project objectives	A fundraising project should have clear objectives including the project purpose, target recipients, and desired outcomes.
	Financing goal	The project should specify the minimum target funding amount that can mobilize the project, and the maximum amount required by the founders.
	Elements of a good project	Elements of a good project should include the following: 1. The project should be rich in content, providing information about the project to potential backers in the forms of text, pictures, and videos.
		2. The content of the project in the presentation must be creative to impress the donors and match the target audience. 3. The type of project must be consistent with the social context, attitudes, beliefs, religions, and urgent social issues that require funding.
	Number of partners	The project should include a number of affiliate networks or well-known charities or individuals involved in fundraising.
	Project duration	The project should have a funding period that is appropriate for its objectives. The funding should not be too long as this may cause backers to feel distrust in the implementation of the project and the ability of founders to achieve project objectives in a timely manner.
	Borderless fundraising	In the future, fundraising projects should not be limited by geography in raising donations.
	Rewards	The project should provide rewards or incentives to Backers to encourage donations.

### Backers

Scenario “Backers in the future should be loyal to the platform and happy with projects they have supported. The platform should be transparent in its fundraising activities, such that donors can evaluate and track the projects’ performance over time.”

To succeed in fundraising, website providers and founders must consider the following three main factors related to backers: motivation, satisfaction and follow-up as shown in Table 11.

**Table 11:** The factors and details of the composition of the backers group.

Factor	Attributes	Description
Motivation	Incentives	The backer will donate money to a particular project whose objectives are of interest to them, relevant to current events, creates a positive impression, has a reward, or if they are a follower of the founder or platform.
	Platform loyalty	Fundraisers often donate money to platforms that they have donated to before, especially when they know their supported projects have been successful in creating a positive impact in their community or larger society. Therefore, it is crucial to publicize project results through the platform to engage backers and let them know their support made a difference to communities and society in accordance with the defined objectives of the project, and that the platform created an environment where founders are accountable for their projects.
	Attitudes and beliefs	Backers should understand the attitudes, beliefs, religions, and fundraising ethics of each region.
	Knowledge of technology	Backers should educate themselves about technologies for fundraising donations, such as cryptocurrency donations and online fund transfer systems.
	Tax deduction	Backers can apply their donations as tax deductions.
Satisfaction	Successful project	Backers often choose to donate to projects of fundraisers with a proven track record, which may influence them to donate to their current projects.
	Happiness	Backers often choose to donate money to projects that give them happiness. If backers have been satisfied with the outcomes of their donations in the past, they may convert from a free platform user to a fee-paying premium user.
	Participation in sponsor activities	Backers often choose to donate to projects that involves them in fundraising activities and highlights their contributions.
	Word-of-mouth	Current backers often recruit other donors to projects that have impressive presentation materials and publicity because it makes them want to be a part of it.
Follow-up	Evaluate the project and Founders	Backers want to assess the project and founders’ performance in the raising and application of funds. A point system for all projects and founders on the platform can help backers easily and consistently assess and compare performance and help with their future donation decision-making.
	Project reports	Backers want to be notified on the performance of the project they have supported after its completion in report form.

An attribute with less than 70% relevance score is Economic Condition under the Motivation category, which could impact backers’ income and ability to donate to various causes.

### Legal

Scenario "In the future, there must be international laws and regulations preventing exploitative and illegal activity on donation-based crowdfunding platforms. Furthermore, there should be an increased emphasis on building an understanding of the ethical and religious contexts of each country through online media.”

The legal aspect can be divided into 2 factors: laws and regulations, with detailed explanation shown in Table 12.

**Table 12:** The factors and details of the composition of the legal group.

Factor	Attributes	Description
Laws	Contract law/ a civil code	Civil code contract law
	Tax law	Tax law
	A law regulating charities and public fundraising	Laws protecting charities and public fundraising
	Consumer protection law	Consumer protection law.
	Data protection law Criminal law (cyber-attack)	Data protection law. Criminal fraud laws, Cyber-attack.
	Laws controlling licenses to raise funds	Laws governing fundraising licenses.
Regulations	Anti-money laundering	Anti-money laundering regulations
	Counter terrorist	Terrorism funding prevention regulations

## Discussion

Assuming pandemics are likely to recur in the future, we explored components and factors that will result in successful donation-based crowdfunding activities. We collected information from existing literature and in-depth interviews with experts based on the EFR technique and principles of the Futures Wheel. This led us to propose guidelines for a successful and sustainable donation-based crowdfunding model that can withstand future pandemics based on stakeholder groups as follows.

### The website providers group

#### System development

To improve the transparency and trustworthiness of donation-based crowdfunding platforms, we find that Blockchain technology should be employed as it can enhance security and reliability for monetary transactions without need for intermediaries [31,37]. Blockchain is a transparent and traceable technology that increases the credibility of the system’s development and provides security, as it will allow donors to track fundraisers’ management of resources, including procurement activities. Further, Blockchain technology has no border limit. Donors can donate through Blockchain without having to convert their local currency to any other currency. Blockchain also allows two unrelated parties to safely exchange and share information. This technology is therefore suitable for online transactions, especially in finance where transactions can be made conveniently, quickly, securely, and most importantly, economically through reduced costs. The idea of transactions without borders is associated with research conducted by Weking, et al. [38] & Chen and Bellavitis [39], which proposed a virtual fundraising model using Blockchain technology such that donors can donate in digital currency. Furthermore, applying Blockchain-based business models in platform and strategy development is consistent with Tonnissen, et al. [40], which suggest that the utilization of Blockchain technology in business can bring about a stakeholder ecosystem that creates value for all users and enable business partners to create their own revenue, simplifying transactions and providing credibility in doing business [38,39]. This can impact business security, increase decentralization of financial businesses and remove restrictions caused by borders, essentially acting as a new landscape for innovation, showcasing the benefits of a transparent and decentralized business model.

A potential drawback of using Blockchain is that transactions are irreversible due to the immutable nature of Blockchain records. This means incidents of sending incorrect amounts of funds or sending funds to incorrect recipients can not be canceled. However, refunds may be possible if the recipient is a known and trusted party. Website providers should carefully consider which Blockchain structure is most suitable for their objectives. The three common structures are private, consortium and public [41]. This research considers two scenarios for canceling Blockchain transactions in a donation crowdfunding setting as follows: 1) Backers wish to reverse donations made, and 2) Founders wish to terminate their projects.

It is uncommon that backers would wish to reverse contributions already made, as these payments are charitable rather than commercial. None of the current platforms provide refunds at present. However, website providers may still consider utilizing a smart contract to set conditions for refunds, such as a 7-day grace period for refund requests. Alternatively, in the case where founders wish to terminate their projects due to their inability to proceed for any reason, they will be able to choose to reallocate funds raised to a different project within the same platform.

Despite challenges surrounding the use of Blockchain, we believe this technology will be beneficial to future donation crowdfunding activities due to its safety, transparency, and accountability. If donations can also be made through digital or cryptocurrencies, perhaps through the utilization of Blockchain bridges [41], donation crowdfunding will become more convenient, instantaneous, and borderless.

An appealing platform should embody the principles of platform business models in analyzing the platform’s people and processes to ensure user-friendliness and attractiveness, which coincides with the research of Joseph, et al. [42], Fehrer, et al. [43], Lee & Kim [44], Shrutika & A. [45], Zhao, et al. [46] Website providers’ should understand the ecosystem of producers and users, identify and analyze interdependent business processes, and specific characteristics of the platform. Navigation should begin on the platform homepage and extend to pages within the scope specified by the platform in order to create a stimulating and positive user experience. This will lead to user loyalty, which will enhance the reputation of the platform. The principles of UX/UI design may be applied to design the end-to-end workflow by giving importance to user experience into the design process. All functions of the platform should be in accordance with Gruen, et al. [47] & Joo [48]. If designers and developers have an understanding of ethnography and apply their technical know-how to design a system that meets the needs of businesses and users, it will result in user satisfaction and, most importantly, a sustainable system.

The website providers’ should provide user guides for new users through a variety of media, including Game-Based Learning techniques in accordance with research by Partovi & Rezavi. [49] which attested that Game-Based Learning is effective in educating, entertaining, and engaging new users with the platform and its fundraising programs. Artificial intelligence (AI) should be employed in developing models for matching projects with likely donors based on their behavior, social activities and interests to increase donations received by the platform, in line with research by Sasaki [22] & Capatina, et al. [50]. Whilst the future use of AI holds potential to influence users’ behavior on a large scale, an immense volume of behavioral and social data, and hence information strategies are required to achieve this.

Finally, the website providers’ should provide founders’ background information and regularly update results of ongoing operations to donors, based on the research by Beaulieu, et al. [17] & Liang, et al. [51]. Reporting can take place through the platform’s blog or other communication channels, as frequent updates to project information are the best way to communicate with donors, maintain transparency, and build trust in the project and platform.

#### Technology

The website providers’ should anticipate integration with new technology arising in the 6G era. Website Providers should prepare to develop new services through social media, mobile applications, and Blockchain technology, based on the research of Liang, et al. [52] & Zhang, et al. [53]. Along with the rise of 6G mobile networks, applications are expected to emerge that are intelligent and highly dynamic. Networks are expected to become ultra-dense and heterogeneous, interconnecting all things with extremely low-latency and high-speed data transmission. AI will enhance the efficiency of intelligent network automation and connect IoT devices with Blockchain technology resulting in security, and most importantly reduce computational costs.

#### Social partner

Social partners should create a network with a business partner, charity foundation, and influencers to promote the platform and increase successful fundraising.

### The founders group

According to research, for future fundraising activities it is very important for founders to have good attributes to provide credibility for their project. Notably, they must have a fundraising license issued by a relevant regulatory body (Donation fundraising license) to reduce the problem of fundraising fraud. Using Blockchain technology in the platform system can alleviate this problem as it can help verify the identity of the parties to transactions, which is in line with the research done by Ramesh, et al. [52]. The Proof-of-Work protocol will be employed as a tool to monitor and record transactions in the network, with smart contract systems implemented to monitor and verify network participants. As fundraising becomes increasingly borderless, founders must be familiar with use of and changes in technology in order to achieve their objectives. Most importantly, Founders must be able to propose their project objectives and information clearly and comprehensively, and engage with donors through impressive promotional efforts to address potential future epidemics or disasters, as stated in the research by Hsieh, et al. [31] & Liang, et al. [51]. Detailed project information will promote a positive attitude towards the project and increase the chances of fundraising success.

### The backers group

The fundraising platform should be continuously improved and updated with attractive projects to encourage donations. The platform should seek to build loyalty to it and its founders by introducing membership tiers. An upgrade from freemium to premium tier should come with privileges determined in line with the research by Gamble, et al. [54]. Consumer satisfaction will also have an impact on willingness to pay, which could be an advanced payment plan (from freemium to premium). Additionally, founders must learn about the culture, beliefs, and religions of each region in which they plan to raise funds, so that they can understand the context of each region’s fundraising project and promote borderless fundraising, which is consistent with the research of Di Pietro and Masciarelli [55]. As future fundraising will become increasingly borderless, founders’ understanding of varying attitudes, beliefs, and religious contexts of each region will have significant influence on garnering project support from backers in different locations. Individuals are more likely to share resources and foster relationships with people living in regions with the same main religion. Furthermore, the founder’s performance should be evaluated and rewarded using a point system, with this information provided to potential backers to help with fundraising decision making. Project performance should be reported to donors across multiple communication channels to promote platform transparency and screen founders for future projects.

### The legal group

Blockchain has the potential to enable cross-regional donation crowdfunding in the near future, but it is not without its challenges. Laws and regulations governing these activities should be amended to facilitate movement of funds, while preserving the intent for donation crowdfunding by preventing exploitation. There may be meetings and seminars to find mutual agreements to create requirements and standards for mutual cooperation between nations. Results of research shows that it is important for each stakeholder to take into account ethical considerations in the future era of fundraising. This is therefore absent from the legal group, which may contradict the initial conceptual framework that the researcher studied in the study done by Beaulieu, et al. [17], where legal and ethical factors were grouped under the same topic.

### The implication of factors on current donation crowdfunding platform

We have summarised desirable platform attributes from the discussion above and identified whether they are present in popular crowdfunding platforms: JustGiving, GoFundMe, and Taejai. We focus on attributes that affect end-users. Show in Table 13.

**Table 13:** Comparison of three crowdfunding platforms based on attributes that affect end-users.

Factor	Attributes	JustGiving	GoFundMe	Taejai
System development	Platform supports worldwide donations	○	○	○
	Attract attention	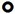	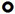	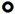
	Ease of use	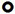	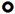	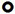
	Instructional media for users	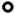	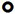	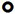
	Digital currency transfer	○	○	○
	Access to founders’ information	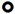	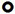	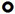
	Use of AI for customized user experience	○	○	○
	Project reporting	●	●	●
	Flexible exchange rate	●	●	●
Technology	Social media support	●	●	●
	Communication tools	○	○	○
	Blockchain	○	○	○
	Support for cryptocurrencies	○	○	○
	Web portal	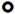	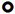	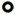
Social partners	Business partners	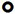	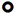	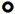
	Charities	●	●	●
	Celebrities and influencers	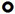	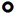	○
Founder experience	Success profile	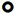	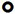	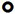
Founder characteristic	Trustworthiness	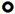	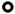	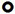
Project/Campaign	Project objectives	●	●	●
	Financing goal	●	●	●
	Elements of a good project	●	●	●
	Number of partners	●	●	●
	Project duration	●	●	●
	Borderless fundraising	○	○	○
	Rewards	●	●	●
Motivation	Tax deduction	●	●	●
Follow-up	Evaluate the project and founders	○	○	○
	Project reports	●	●	●

● Attribute exists on platform.

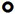
 Attribute partially exists on platform.

○ Attribute does not currently exist on platform.

Our analysis finds that current platform designs are simple and convenient. Founders may start fundraising by merely creating a project on the platform, stipulating the target amount, describing their goals through a written description and multimedia, and obtaining approval from the platform. Subsequent to approval, Founders can easily manage their projects through the platform dashboard. Each platform has its own conditions and limitations, most notably in terms of geography, currency, and applicable laws and regulations. Therefore, these platforms still have room to grow in the future.

We believe that the introduction of Blockchain technology can create a more decentralised information management system and facilitate borderless operations through the utilization of digital currency. This in turn has the potential to increase transparency, safety and convenience, and reduce transaction costs compared to existing payment methods. Currently, most payment fees are determined by the individual platform. AI can be integrated into the platform’s Customer Relationship Management (CRM) system to learn the behaviors of individual backers and provide personalised campaign recommendations. The platform needs to be compatible with popular technology in order to maximise access to users. Cooperation with social media and e-commerce platforms can allow access to audiences and tools such as fundraising through a Live video, with donations being shown on screen real time. Consequently, we believe that platforms should use Game-Based Learning to attract and retain users to the platform. At present, most platforms have not focused on platform appearance and format. Using Game-Based Learning can be an engaging way to build loyalty and differentiate one platform from another. Finally, the current web portal are undergoing ongoing enhancements, as the numerous platforms are still siloed and there are concerns over each country’s rules and regulations. When website providers expand and connect their platform with a global network of business partners and adhere to standardized protocol, the web portal will be able to give comprehensive support.

Though founders’ profiles and past project information are currently available, the platform should also clearly display any fundraising licenses they hold and ratings from past backers to improve their credibility to potential donors. In concert with this, platforms should provide backers with an opportunity to rate their experience with founders they have supported in terms of project execution and results. Ratings collected over time can provide a more complete picture of a founder’s capabilities and direct charitable funds to the most effective use.

On the legal side, we hope to see the development of new laws and regulations that will support borderless fundraising.

### Conclusions

We conducted this study using the principles of Futures Research. We interviewed stakeholders and experts, used the Futures Wheel methodology to visualize the future and extract key success factors for donation crowdfunding based on each stakeholder group, and conducted cross-impact analyses. Finally, the future scenarios were determined. Our research results show the key factors of success are as follows; 1) For the websites providers group the three factors are system development, technology, and social partner 2) For the founders group the three factors are founder experience, founder characteristic, and project or campaign 3) For the backers group, the three factors are motivation, satisfaction, and tracking 4) The legal group consisted of two factors, being law and rules. We used the scenarios for each group of stakeholders as a guideline for developing a conceptual framework for the future development of donation crowdfunding framework.
