# Correction: The future of digital donation crowdfunding

**DOI:** 10.1371/journal.pone.0335353

**Published:** 2025-10-23

**Authors:** Siriphong Sirisawat, Pattanaporn Chatjuthamard, Supaporn Kiattisin, Sirimon Treepongkaruna

In the Methods subsection of the Data collection process, there is a missing sentence. The correct sentence is: As our study is human subject research, we sought required approval from the Institutional Review Board (IRB) to conduct this research. After receiving this approval, we began collecting data through interviews with stakeholders and experts, taking notes and voice recordings. Interview lengths range from 45 to 60 minutes. We then utilised data collected to create a Futures Wheel and conducted a cross matrix scenario analysis. The certificate details are as follows: COA No. MU-CIRB 2020/163.1410.
